# Auditory Processing Differences Correlate With Autistic Traits in Males

**DOI:** 10.3389/fnhum.2020.584704

**Published:** 2020-10-07

**Authors:** Simge Aykan, Emre Gürses, Suna Tokgöz-Yılmaz, Canan Kalaycıoğlu

**Affiliations:** ^1^Department of Physiology, School of Medicine, Ankara University, Ankara, Turkey; ^2^Department of Audiology, Faculty of Health Sciences, Hacettepe University, Ankara, Turkey; ^3^Department of Audiology, Faculty of Health Sciences, Ankara University, Ankara, Turkey; ^4^Audiology, Speech and Balance Diagnosis and Rehabilitation Center, School of Medicine, Ankara University, Ankara, Turkey

**Keywords:** autistic traits, sensory sensitivity, sex differences, visual evoked potentials, auditory evoked potentials

## Abstract

Autism spectrum disorder (ASD) has high prevalence among males compared to females but mechanisms underlying the differences between sexes are poorly investigated. Moreover, autistic symptoms show a continuity in the general population and are referred to as autistic traits in people without an ASD diagnosis. One of the symptoms of ASD is sensory processing differences both in sensitivity and perception. To investigate sensory processing differences in autistic traits, we examined auditory and visual processing in a healthy population. We recruited 75 individuals (39 females and 36 males, mean age = 23.01 years, *SD* = 3.23 years) and assessed autistic traits using the Autism Spectrum Quotient, and sensory sensitivity using the Sensory Sensitivity Scales. Sensory processing in the visual domain was examined with the radial motion stimulus and the auditory domain was assessed with the 1,000 Hz pure tone stimulus with electroencephalography-evoked potentials. The results showed that the auditory sensitivity scores of the males (*r*_aud_ (34) = 0.396, *p*_aud_ = 0.017) and the visual sensitivity scores of females were correlated with autistic traits (*r*_vis_ (37) = 0.420, *p*_vis_ = 0.008). Moreover, the P2 latency for the auditory stimulus was prolonged in the participants with a higher level of autistic traits (*r*_s_ (61) = 0.411, *p* = 0.008), and this correlation was only observed in males (*r*_s_ (31) = 0.542, *p* = 0.001). We propose that auditory processing differences are related to autistic traits in neurotypicals, particularly in males. Our findings emphasize the importance of considering sex differences in autistic traits and ASD.

## Introduction

Although sex differences at behavioral and neural levels have long been recognized (Cahill, 2006), information concerning the measures of neural processing differences related to sex is still scarce. One proof of sex related differences in brain is that the prevalence of neuropsychological disorders differs between men and women. In the literature, there are many examples of diseases, in which the number of females and males are not equal. A potential difference in the pattern of sex bias is the preponderance of early onset neurodevelopmental disorders in males, which might indicate biological differences between sex. For example, major depressive disorder, anxiety, and Alzheimer’s disease have a higher female ratio while attention deficit/hyperactivity disorder, autism spectrum disorder (ASD), dyslexia, and early onset schizophrenia are seen at a higher rate among men (McCarthy et al., 2012).

Autism spectrum disorder is an early onset neurodevelopmental disorder characterized by communication and social skill deficiencies (American Psychiatric Association, 2013). Similar to all neurodevelopmental disorders, ASD has high male prevalence at a ratio of 4:1 (Werling and Geschwind, 2013). This difference in sex prevalence has been recognized since Asperger made his first observations (Bryson and Smith, 1998; Fombonne, 2003, 2009). The current male-bias in ASD raises the question of how sex prevalence might be relevant to the underlying mechanisms of this disorder. There are many possible explanations ranging from the differences in the mechanisms involved in males and females to the presence of protective mechanisms in the latter or vulnerability mechanisms in the former (Halladay et al., 2015; Lai et al., 2015; Ferri et al., 2018; Zhang et al., 2020).

Sensory processing consists of the steps of perception, modulation, and integration of sensory information (Ayres, 1972). Perception refers to noticing sensory experiences while sensory modulation encompasses both a neurophysiological process and behavior (Brown et al., 2019). One of the aspects of sensory modulation is sensory sensitivity, ranging from hyposensitivity to hypersensitivity for environmental stimuli. Hypersensitivity refers to an exaggerated behavioral response to stimuli, while hyposensitivity refers to the insufficiency or absence of a response to any kind of stimuli (Baranek, 2002; Baranek et al., 2006). These sensitivity differences can be related to primary sensory processing or higher cognitive processes, or both (Brown et al., 2019). Various studies have shown differences in sensitivity for taste, pain/touch, vision and olfaction between males and females in general (Prutkin et al., 2000; Greenspan et al., 2007; Loyd and Murphy, 2014). In visual processing, males have significantly greater sensitivity for fine detail and rapidly moving stimuli, while females exhibit better color discrimination (Abramov et al., 2012). For auditory stimuli, pure-tone hearing sensitivity declines faster in males at most frequencies (Rosenhall, 2003).

One of the symptoms of ASD is atypical sensory processing. Sensory sensitivity has been shown to vary between individuals with ASD and the prevalence of sensory sensitivity for this disorder ranges from 69 to 96% (Baranek et al., 2006; Geschwind, 2009; Klintwall et al., 2011; Marco et al., 2011). Sensory abnormalities in individuals with ASD were included as a diagnostic criterion in the latest version of The Diagnostic and Statistical Manual of Mental Disorders (DSM) (American Psychiatric Association, 2013). The most altered modalities are visual, auditory, and tactile senses (Tavassoli et al., 2014, 2016; Fernández-Andrés et al., 2015). Despite these known differences between sexes, studies related to sensory processing in ASD have not focused on these differences.

In addition to sensitivity differences, perception differences are also present in ASD. With its high temporal resolution and recording of activity of neural ensembles, electroencephalography (EEG) is mostly the method of choice in sensory perception studies. Studies have documented atypical processing of auditory and visual input with EEG (for a review; Marco et al., 2011). Atypical auditory processing is revealed from basic acoustic properties, such as slower click-evoked auditory brainstem response relative to the typical development of children (Rosenhall et al., 2003) and poorer tracking of pitch contours (Russo et al., 2008) to more complex information, including prosody and speech perception in noise (for reviews; Haesen et al., 2011; O’Connor, 2012). Among auditory stimuli, 1000 Hz pure-tone burst sound is widely used in ASD research. Although most research showed a difference between ASD individuals and controls, results are conflicting as some studies found shorter latencies (Martineau et al., 1984; Ferri et al., 2003) while others found longer latencies (Oram Cardy et al., 2008; Roberts et al., 2010). For visual stimuli, ASD individuals tend to be poor at processing motion and faces (for a review; Yamasaki et al., 2017). The magnocellular pathway plays an important role in detecting motion and processing global structure. To investigate global motion processing, coherent motion stimuli such as radial optic flow and horizontal motion have been used. Findings have indicated the selective impairment of radial movement perception, in adults with ASD (Yamasaki et al., 2014; Van der Hallen et al., 2019). Radial orbital flow is a type of complex motion characterized by multidirectional movement with depth, which is also the basis of action-related information, including biological motion (Kuba et al., 2007).

Another aspect of ASD is its spectrum of characteristics. Since individuals with ASD exhibit a varying degree of severity of symptoms, autistic traits in the general population also has a Gaussian distribution signifying varying degrees of autistic traits (Baron-Cohen et al., 2001; Ruzich et al., 2015). Therefore, ASD/autistic traits could be considered as a continuous variable. In such a case, we would expect sensory processing differences between sex for ASD, as well as a relation of sensory processing differences with autistic traits in the general population. In accordance with our second assumption, studies have shown sensory processing differences in healthy samples (Robertson and Simmons, 2013; Horder et al., 2014; Tavassoli et al., 2014; Mayer, 2017).

Based on these assumptions, in the present study, we investigated sensory processing differences related to autistic traits in neurotypicals and possible sexual dimorphism in this process. For this purpose, we used the Sensory Sensitivity Scales (SeSS) (Aykan et al., 2020) for the sensory sensitivity measurement, radial motion as the visual stimulus, and 1,000 Hz pure-tone burst as the auditory stimulus for the sensory perception assessment since they seem to be impaired in ASD (Haesen et al., 2011; O’Connor, 2012; Yamasaki et al., 2017). Responses to stimuli were analyzed with EEG. We assessed (i) relation of autistic traits with sensory sensitivity in visual and auditory domains, (ii) whether there was a difference between females and males for sensory sensitivity, (iii) whether the differences in subjective assessment reflected EEG-evoked potentials, and (iv) the relation of evoked potentials with autistic traits and effect of sex on this relation.

## Materials and Methods

### Study Design and Measurement Tools

The study was based on two sessions. In the first session, the participants completed forms which gave demographic information and health status. Suitable participants for the study were examined with the pure-tone audiometry and Snellen visual acuity tests.

The pure-tone audiometry test was performed in a sound-isolated booth according to the ANSI 1996 standards. The measurements were performed by an experienced audiologist using the Interacoustics AC40 (Assens, Denmark) clinical audiometer and Telephonics TDH-39 (Telephonics, Farmingdale, NY, United States) supra-aural headphones. Pure-tone hearing thresholds were assessed between 125 Hz and 8 kHz (in ½ octave steps) for both ears in compliance with the modified Hughson–Westlake procedure (Carhart and Jerger, 1959). The Snellen chart (Snellen, 1862) consisting of letters arranged according to the Snellen principle was used to determine visual acuity. Each participant was asked to read the letters shown at a distance of 6 m, using the left and right eyes separately (with glasses if used). A vision of 6/6 was considered normal. Individuals with normal or corrected-to-normal vision and having hearing within normal limits were invited to the second session of the study.

In the second session, the participants completed the Turkish-language version of the Autism Spectrum Quotient (AQ) (Baron-Cohen et al., 2001; Kose et al., 2010) and the visual and auditory SeSS (Aykan et al., 2020).

Autism Spectrum Quotient is a 50-item self-report measure of preferences and tendencies in the participant’s daily life. The maximum score is 50 points, and higher scores indicate higher levels of autistic traits. The test measures variability in attention switching, social skills, attention to detail, communication, and imagination. SeSS measure sensory sensitivity in visual, auditory and somatosensory domains without interference from social and emotional features in typically developed adults. Each scale is independently evaluated. In the current study, the visual and auditory scales were used. Construct validity were good both in the visual (CFI = 0.973, TLI = 0.965, and RMSEA = 0.075) and auditory (CFI = 0.943, TLI = 0.927, and RMSEA = 0.074) domains. The categories were internally consistent (α_*vis*_ = 0.86, α_*aud*_ = 0.79). The scales were comparable as both consisting of 10 items with a total score from 0 to 50 points for each (Aykan et al., 2020). The EEG of each participant was recorded separately for visual and auditory stimuli.

### Participants

A total of 75 (39 females and 36 males, mean age = 23.01 years, *SD* = 3.23 years), undergraduate or graduate students participated in the study. The inclusion criterion was being aged between 18 and 30 years and pure-tone hearing thresholds of 20 dB or better at frequencies of 250–8000 Hz, and the exclusion criteria were auditory impairment, uncorrected visual impairment, a diagnosed neuropsychiatric disorder, and taking neuropsychiatric medication. The study was approved by the Ethical Committee of Ankara University School of Medicine and designed according to the principles of the Declaration of Helsinki.

Autistic traits (mean score = 17.35, *SD* = 5.25, range [8; 32]), and the visual and auditory SeSS scores were calculated (Visual Sensitivity: mean score = 26.05, *SD* = 7.53, range [12; 46]; Auditory Sensitivity: mean score = 29.07, *SD* = 6.61, range [14; 43]).

### Electrophysiological Data Collection and Processing

#### Visual and Auditory Stimuli

The visual stimulus was a radial expansion motion stimulus in low-contrast (10%) concentric circles with sinusoidal luminance modulation (Kremláček et al., 2004). A temporal frequency of 5 cycle/second was kept constant over the whole stimulus field. A black square in the middle of the screen was used as a fixation point. The stimuli had 200 ms motion, followed by a 1,000 ms interstimulus interval stable image. Total stimulus repetition was 60.

The auditory stimulus was a 1,000 Hz tone burst and was presented binaurally via Hosiden DH-05-S circumaural headphones (Hosiden Electronics, Japan) at a level of 70 dB SPL. A gray screen with a white “+” sign was shown to prevent eye movement during recording. The duration of the test stimuli was 200 ms, followed by a 1,000 ms interstimulus interval. The stimulus was presented 60 times.

All stimuli were presented on a 22-inch computer monitor (AOC International GmbH, Germany) with a 60 Hz refresh rate from a 70 cm observing distance. For the presentation of stimuli, an Intel (R) Core (TM)2 Quad CPU Q8300 2.50 GHz computer; ATI Radeon HD 3400 Series graphic card and an Eugene Gavrilov kX 10k1 Audio (3550) sound card were used. The software used to present the stimuli was Psychtoolbox-3.0.8 (Kleiner et al., 2007) and Matlab-R2008a (MathWorks Inc., United States).

#### Data Collection

The EEG data were recorded using BrainVision (Brain Products GmbH, Germany) with 30 Ag-AgCl active electrodes mounted on an elastic cap using the extended 10–20 system. During recording, all electrodes were referenced to the FCz electrode. Eye movements were monitored using vertical and horizontal electrooculography (EOG) electrodes attached to the external canthi and the supraorbital regions of the right eye. Both EEG and EOG signals were digitally amplified and sampled at 1,000 Hz. All electrode impedances were kept below 10 kΩ.

#### Data Processing

The data were analyzed using BrainVision Analyzer 2.1 software (Brain Products GmbH, Germany). Initially, the raw EEG data were passed through a 0.5–30 Hz band pass filter. The channels were re-referenced offline to the digital average of mastoid electrodes. The data epochs consisted of 200 ms before and 500 ms after the stimulus onset. Eye blinks and artifacts were visually examined, and segments containing artifacts were removed. Subjects with total number of segments less than 40 epochs were excluded from further analysis, data from 33 males and 31 females were analyzed for visual stimulus. Data from 34 males and 32 females were analyzed for auditory stimulus. The mean number of segments after the removal of artifacts was 50.31 (*SD* = 6.29, range [40; 60]) for the auditory stimulus and 48.50 (*SD* = 6.54, range [40; 60]) for the visual stimulus. Baseline correction was applied to 100 ms prior to the stimulus presentation, where the average voltage level in the defined region corresponds to the new zero point of the segment values.

**FIGURE 1:**
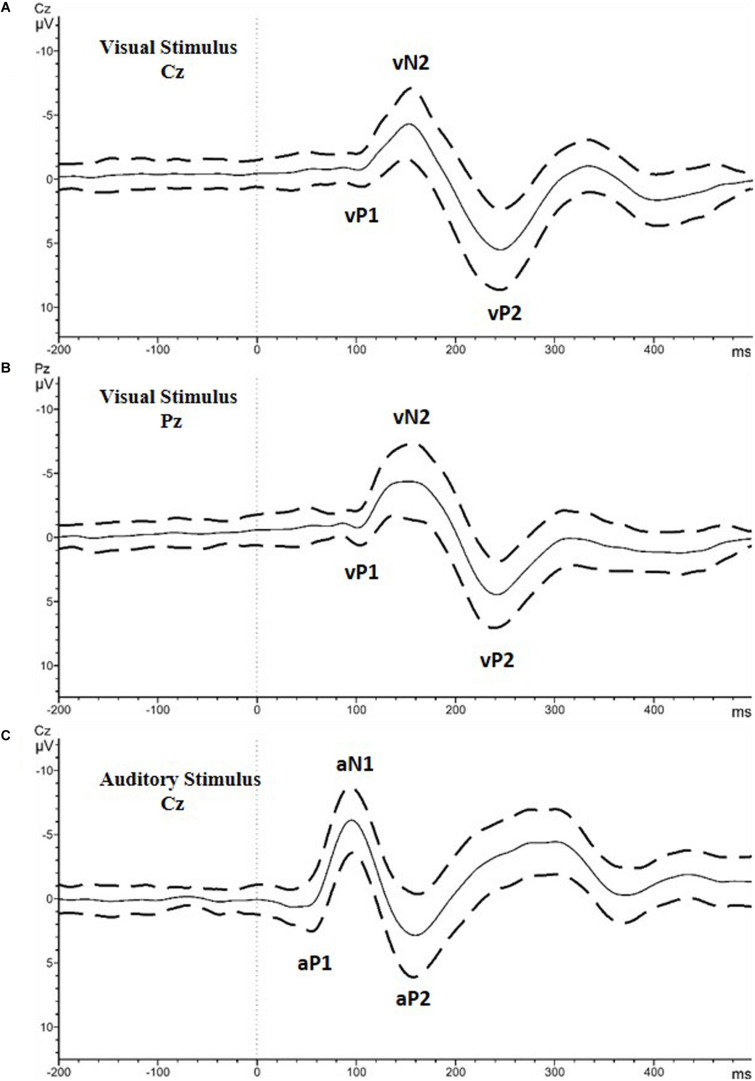
Grand average of all the included participants with the standard deviation values. **(A)** Visual stimulus recorded at Cz electrode. **(B)** Visual stimulus recorded at Cz electrode. Analyzed peaks are marked as vP1, vN2, and vP2. **(C)** Auditory stimulus recorded at Cz electrode. Analyzed peaks are marked as aP1, aN1, and aP2.

For the visual stimulus, the P1, N2, and P2 peaks were computed for the Cz and Pz channels (Figures 1A,B shows the visual evoked potential (VEP) peaks with a lower case “v”; vP1, vN2, vP2). All the amplitude values were calculated as a peak to peak difference of the target peak and the former peak. The amplitude of vN2 was calculated as the peak to peak difference between vP1 and vN2 at the Pz electrode. For vP2, the amplitude was calculated as the peak to peak difference between vN2 and vP2 at the Cz electrode. For both potentials, the latency was calculated as the time from the presentations of the stimulus to the peak point of the potential.

For the auditory stimulus, the amplitude and latency of the P1, N1 and P2 peaks were computed for the Cz electrode (Figure 1C shows the auditory evoked potential (AEP) peaks with the lower case “a”; aP1, aN1, aP2). Similar to the visual potentials, the amplitude of aN1 and aP2 were calculated as the peak to peak difference between the potentials and the former peaks. For both potentials, the latency was calculated as described for the visual stimulus.

### Statistical Analysis

Outliers with a z-score higher than ±3.0 were excluded from the statistical analysis for all the ERP measures, autistic trait and sensory sensitivity scores. Normality was checked using the Shapiro–Wilk test. Variants with normal distribution (autistic trait and SESS scores) were analyzed with parametric tests (Student’s *t*-test, ANOVA and Pearson correlation) while variants with non-normal distribution (ERP measures) were analyzed with non-parametric tests (Mann Whitney-U and Spearman correlation). Direct comparison of correlation coefficents was performed by Meng-Rosenthal-Rubin method (Meng et al., 1992) using R 3.3.3 (R Core Team, 2018). All other statistical analyses were performed using SPSS software version 22.0 (IBM Corp. Released 2011, 2011). An overall 5% type-I error level was used to infer statistical significance. The Bonferroni correction was undertaken when required.

## Results

### Auditory Sensitivity for Males, Visual Sensitivity for Females Is Correlated With Autistic Traits

Shapiro–Wilk test indicated normal distribution of SESS sensitivity scores (*p* < 0.05). The results of the Pearson correlation showed that visual sensitivity and auditory sensitivity scores of SESS were strongly related; *r*(73) = 0.631, *p* < 0.001. Age and auditory sensitivity were not correlated; *r*_aud_ (73) = 0.030, *p*_aud_ = 0.802, but for visual sensitivity, there was a positive correlation *r*_vis_ (73) = 0.266, *p*_vis_ = 0.021. Age was not statistically different between the females and males (*t*(73) = 3.797, *p* = 0.325).

We performed 2 × 2 mixed factor analyses of variance (Mixed ANOVA) including modality (visual and auditory sensitivities) as within factors and sex (female and male) as the between factors. Our result indicated that there was no statistically significant interaction between modality and sex, *F*(1,73) = 0.923, *p* = 0.340, *$\eta_{p}^{2}$* = 0.012. The main effect of modality showed a statistically significant difference in sensitivity scores *F*(1,73) = 18.376, *p* < 0.001, $\eta_{p}^{2}$ = 0.201. Bonferroni comparison test revealed that Auditory sensitivity score (29.07 ± 6.61) was significantly higher than Visual sensitivity score (26.05 ± 7.53) with mean difference 3.04 (SE = 0.71) points (*p* < 0.001; 95% CI: 1.67–4.424). There was no significant main effect of sex (*p* = 0.770).

**FIGURE 2:**
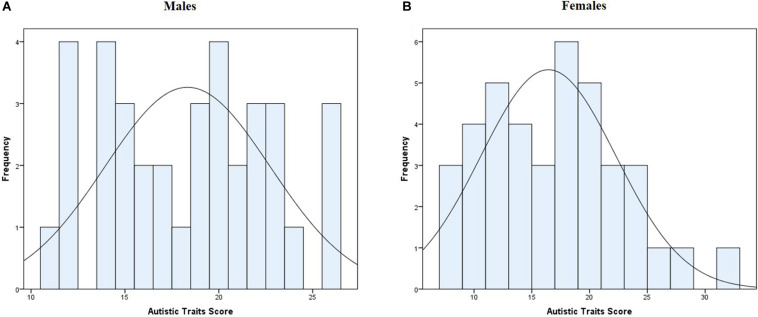
Distribution of autistic traits score panel **(A)** for males, panel **(B)** for females.

Both sensitivities had positive correlations with autistic traits; *r*_vis_ (73) = 0.328, *p*_vis_ = 0.004 and *r*_aud_ (73) = 0.273, *p*_aud_ = 0.018. Autistic traits both in females and males were normally distributed (*W*_*male*_(36) = 0.954, *p*_*male*_ = 0.136; *W*_*female*_(39) = 0.963, *p*_*female*_ = 0.231; Figure 2). Autistic traits were not different between the females (*M* = 16.44, *SD* = 5.84) and males (*M* = 18.33, *SD* = 4.40), *t*(73) = −1.577 *p* = 0.119.

When the correlations were examined for each sex, visual sensitivity was correlated with autistic traits in females (*r*_vis_ (37) = 0.420, *p*_vis_ = 0.008 and *r*_aud_ (37) = 0.202, *p*_aud_ = 0.218) while auditory sensitivity was correlated with autistic traits for males (*r*_vis_ (34) = 0.244, *p*_vis_ = 0.152 and *r*_aud_ (34) = 0.396, *p*_aud_ = 0.017).

### Males Have Longer Latency for Auditory Potentials

At the 1,000 Hz pure-tone auditory stimulus, the correlations between the general hearing level, 1,000 Hz specific hearing level, and AEP were examined, and no significant correlation was found (*p* > 0.05).

For all potentials, latency and amplitude compared between the females and males (Table 1), and the latter (Mdn_*aN1*_ = 100.50, Mdn_*aP2*_ = 167) had significantly longer auditory aN1 and aP2 latency than the former (Mdn_*aN1*_ = 92, Mdn_*aP2*_ = 158) after the Bonferroni correction for eight comparisons; *U*_*aN1*_ = 254.0, *p* = 0.008, *U*_*aP2*_ = 305.0, *p* = 0.024.

**TABLE 1:** Descriptives and comparison of evoked potentials for the participants according to sex.

	**Female (*n* = 27–32)**					**Male (*n* = 33–34)**						
	
**EEG Measure**	**Min**	**Max**	***M***	***SD***	**Median**	**Min**	**Max**	***M***	***SD***	**Median**	***U***	p*
Auditory N1 Latency	75	105	92.45	6.562	92	80	117	99.30	8.24	100.50	254.0	**0.008**
Auditory P2 Latency	135	180	158.69	10.985	158	140	192	168.06	12.22	167	305.0	**0.024**
Auditory N1 Amplitude	2.60	13.12	6.76	2.58	6.33	2.72	8.94	6.59	1.51	6.88	432.0	>0.500
Auditory P2 Amplitude	4.66	22.63	11.23	4.48	9.38	3.90	14.99	8.45	2.60	7.98	306.5	0.072
Visual N160 Latency	124	191	162.44	17.72	163	130	192	158.47	18.02	156	362.5	>0.500
Visual P240 Latency	213	263	238.96	12.64	240	214	264	243.44	12.46	245	335.0	>0.500
Visual N160 Amplitude	0.29	10.80	4.76	2.74	5.15	0.98	10.52	5.59	2,64	5.75	358.0	>0.500
Visual P240 Amplitude	6.40	23.96	11.65	4.27	10.37	5.42	17.72	9.37	2.90	8.66	281.0	0.176

*p is the Bonferroni-corrected value. Bold text indicates a statistically significant difference with a *p*-value less than 0.05.

### For Males, P2 Latency for Auditory Stimulus Prolongs With Higher Autistic Traits

Auditory sensitivity was not correlated with auditory potentials, and visual sensitivity was not correlated with visual potentials (*p* > 0.05). Autistic traits and aP2 latency for the auditory stimulus were moderately correlated (Table 2) after the Bonferroni-corrected value for eight comparisons *r*_*s*_ (61) = 0.411, *p* = 0.008. When the correlation was reanalyzed for sex, the autistic traits were correlated with the aP2 latency for the males [*r*_*s*_ (31) = 0.542, *p* = 0.001] while no correlation was observed for the females [*r*_*s*_ (30) = 0.266, *p* = 0.141]. Direct comparison of correlation coefficients between males and females showed no statistically significant difference *z* = −1.172, *p* = 0.240.

**TABLE 2:** Means, standard deviations, and correlations for the EEG parameters and autistic traits.

**Variable**	**M**	**SD**	**1**	**2**	**3**	**4**	**5**	**6**	**7**	**8**
1. AQ Score	17.35	5.257								
2. Auditory N1 Latency	95.98	8.183	0.137							
3. Auditory P2 Latency	163.45	12.471	0.411**	0.412**						
4. Auditory N1 Amplitude	6.671	2.067	0.158	0.147	0.010					
5. Auditory P2 Amplitude	9.779	3.858	–0.008	–0.302	–0.046	0.372*				
6. Visual N160 Latency	160.29	17.842	–0.133	–0.014	–0.059	–0.227	0.097			
7. Visual P240 Latency	241.39	12.641	0.195	0.231	0.145	0.161	–0.044	0.065		
8. Visual N160 Amplitude	5.213	2.696	0.056	0.042	0.095	0.160	–0.008	–0.241	–0.010	
9. Visual P240 Amplitude	10.420	3.746	0.085	–0.146	–0.044	0.345	0.293	–0.282	0.039	0.083

* indicates *p* < 0.05, **indicates *p* < 0.01 after Bonferroni correction.

## Discussion

Within the scope of this study, we investigated the presence of sensory processing differences in males and females in relation to autistic traits. Significant positive correlations were found between sensory sensitivity scores and autistic traits. When analyzed based on sex, autistic traits were significantly correlated with auditory sensitivity scores in males and visual sensitivity scores in females. The second aim of this study was to investigate sensory perception differences related to autistic traits, and we showed that the latency of the aP2 auditory potential was positively correlated with autistic traits; moreover, this correlation only applied to the males.

Initially, we showed that both visual and auditory sensitivities measured by SESS were correlated with autistic traits in neurotypicals. Although there was no difference in the mean scores between sex, the correlation was observed in the visual domain for females and the auditory domain in males. In support of our finding, previous studies showed a correlation of sensory sensitivity with autistic traits in healthy adults (Robertson and Simmons, 2013; Horder et al., 2014; Tavassoli et al., 2014; Mayer, 2017). Secondly, the mean scores of men and women did not differ for the visual and auditory domains similar to the literature (Robertson and Simmons, 2013; Tavassoli et al., 2014); however, those studies did not evaluate the relation of autistic traits and sensory sensitivity specific to the sex of the participants. Horder et al. (2014) analyzed the relation of autistic traits and sensory sensitivity specific to sex and did not find any difference in the total score of sensory sensitivity; they did not evaluate domain-specific sensitivity.

The second outcome of the study was the relation of sensory perception with autistic traits based on evoked potentials. We showed that auditory-evoked potential latency was correlated with autistic traits, and this correlation was preserved only in males. The latency or amplitude of VEPs was not related to autistic traits, and there was no difference in VEPs between sex. The stimulus used to acquire VEPs was a low-contrast motion stimulus that mainly examined the magnocellular system and dorsal stream of the visual pathway. With regard to whether impairments were observed in motion processing, there are controversial results in the literature. High-risk infants showing familial risk exhibited atypically high luminance contrast sensitivity (McCleery et al., 2007) while adolescent siblings of ASD had elevated chromatic contrast sensitivity (Koh et al., 2010). In addition, a decreased efficiency of motion processing during luminance stimuli was shown to be present both in ASD individuals and in their siblings (Koh et al., 2010). Using contrast sensitivity to discern the motion direction of luminance gratings, one previous study reported reduced sensitivity in adolescents with ASD only for those with a history of language delay (Takarae et al., 2007) while another determined a typical performance in adolescents with ASD (Bertone et al., 2003). In another study, the authors compared individuals with high and low AQ and found mixed results for motion coherence performance. They also observed a delayed magnocellular VEP at high contrast for the high AQ group (Sutherland and Crewther, 2010). Different from that study, our stimulus was low-contrast radial motion, and we investigated the relationship in a dimensional way, rather than by categorical grouping. Overall, our results are supported by a part of the literature that shows no difference in the magnocellular system.

In terms of the auditory stimulus, we showed that the prolonged latency of aP2 was related to autistic traits across the whole group, but this relation was preserved only in males. Furthermore, the latency values of both auditory potentials were higher for the males independent of autistic traits. This finding is compatible with the prolonged latency being related to autistic traits only in males. Similar to our study, longer aN1 and aP2 latency in males but also higher amplitude for females have been documented (Jaworska et al., 2012). However, this amplitude difference for females was obvious at higher loudness levels of sound (100 dB). In another study, it was shown that the sex difference for amplitude appeared after 90 dB SPL (Hensch et al., 2008). We used 70 dB SPL, which might explain the difference between studies regarding amplitude. Our finding of prolonged latency in males might also contribute to the understanding of the vulnerability of males to autism or the idea of protective factors for females in this disorder. Human studies showed M100 latency delay with MEG, which is similar to the aN1 potential of EEG in children with ASD (Gandal et al., 2010; Roberts et al., 2010). Furthermore, while the M100 peak latency decreased with the increasing age in typically developing children, latency was stable in children with ASD in two studies consisting only males (Gage et al., 2003) and mostly males (3 females/13 males; Stephen et al., 2017). In contrast to our study, Ferri et al. (2003) showed shorter in N1 latency for ASD subjects with intellectual disability comorbidity. In another study, latencies were shorter for both N1 and P2 potentials for ASD subjects while there was no difference for amplitudes (Martineau et al., 1984). Here 13 children out of 18 subjects were failed to develop proper speech. In the last two studies mentioned, findings contradicting with our study might be explained with the severity of the malfunction, as our subjects are neurotypicals. In addition to human studies, animal models of ASD show prolonged aN1 and aP2, especially when epilepsy is present. In the CDKL5 model, where no early onset seizure was present, only the aP2 latency was prolonged (Wang et al., 2012). Consistent with our findings in human males, all animal models were consisted of only male animals.

Auditory-evoked potentials represent the population of neurons firing synchronously in response to sound. Auditory-evoked potentials we examined, aN1 and aP2, are generated in the primary auditory and association cortices, respectively (Modi and Sahin, 2017). Evoked potentials provide information about early functional changes of the sensory neural networks, which are sometimes recognizable prior to any detectable changes observed by imaging techniques. At this point, we might consider epilepsy as a big deviation from the normal functioning of neural networks. If there was such a severe insult on the networks, it would be possible to observe a deviation even at the lower level (primary sensory areas). Since our participants were undiagnosed individuals, we can consider them as having a mild form of the disorder, in which it would be unlikely that there could be a severe effect on the network. Thus, we would not expect an epilepsy comorbidity. Moreover, we did not include subjects with epilepsy or any neuropsychiatric disorder.

Since successful social interaction requires the perception of information about other people and the surrounding environment, disturbances to time-sensitive integration of information would affect social skills. Increased evoked potential latency is considered to indicate the slowing of neuronal processing, which should not only be attributed to the measured process but should also be generalized to all networks in the related brain area. If we consider our study specifically from this perspective, we can conclude that the temporal association cortex is affected in males while there is no difference in females. Prolonged AEP might be an indicator of disturbances to networks. In accordance with this, there are a large number of studies showing temporal cortex disturbances in ASD, especially in males (Meresse et al., 2005; Wallace et al., 2010; Pelphrey et al., 2011; Ha et al., 2015; Irimia et al., 2017). The presence of a delay only in males might indicate more disturbances in males or the existence of protective factors in females. In accordance with this, neurodevelopmental disturbances are mostly present in males that might show more severe disturbances in networks. We might consider auditory P2 latency as a biomarker of integrity of temporal networks. This idea is supported by both animal and human studies (for a review; Modi and Sahin, 2017).

One output of this study is pointing out the importance of investigating sex differences in ASD. As the prevalence of males is high and there is a stability in the hormonal cycle, males are preferred both in animal and human studies. The main consequence of this approach is that the proposed mechanism of the disorder mostly explains disturbances in males that would lead to misdiagnosis and/or wrong treatment of females.

Another important result of the study is its contribution to the idea of a continuum of ASD, not only in diagnosed people but in a total population. Our study contributes to the literature of ASD as a continuous trait, in which people are diagnosed if the level of this trait is at the higher end in the overall population. This association seen across the range of AQ scores provides evidence for a dimensional measure of the severity of autistic traits and a dimensional link between sensory abnormalities and ASD traits instead of the common categorical approach. In conclusion, sensory traits could serve as a quantitative dimensional measure of the severity of autistic traits.

## Limitations

The present study has many strengths; however, some limitations should also be acknowledged. The sample has a relatively small size and comes from nearly the same education level. To drive conclusions for the contribution of these to autistic traits in general population our findings should be assessed in a larger population. We were unable to find any relation between the sensory sensitivity scores and evoked potentials. We can explain this as sensory sensitivity having many components that contribute to it, from the physiological to the psychological level. One possibility is that it could be quiet variable that contributes to this in a population. Also, the evoked potentials that we measured might not cover the sensory process that is affected the assessed modalities. Finally, visual-auditory sensitivities and perception need to be investigated with different measurements/stimuli as they are complex processes with so many components. Here, we have presented indicators that sensory processing is impaired in relation to autistic traits, similar to ASD.

## Conclusion

We propose that the auditory processing differences are related to autistic traits, particularly in males relative to females in neurotypicals, a difference that is also observed in ASD. Our findings emphasize the importance of considering sex differences in autistic traits and autism.

## Data Availability Statement

The raw data supporting the conclusions of this article are available from the corresponding author on reasonable request.

## Ethics Statement

The studies involving human participants were reviewed and approved by Ethical Committee of Ankara University School of Medicine. The patients/participants provided their written informed consent to participate in this study.

## Author Contributions

SA: conceptualization, methodology, investigation, and writing – original draft. EG: methodology, investigation, and writing – review and editing. ST-Y: methodology and writing – review and editing. CK: supervision, methodology, and writing – review and editing. All authors contributed to the article and approved the submitted version.

## Conflict of Interest

The authors declare that the research was conducted in the absence of any commercial or financial relationships that could be construed as a potential conflict of interest.
